# Wnt3a Induces Myofibroblast Differentiation by Upregulating TGF-β Signaling Through SMAD2 in a β-Catenin-Dependent Manner

**DOI:** 10.1371/journal.pone.0019809

**Published:** 2011-05-18

**Authors:** Jon M. Carthy, Farshid S. Garmaroudi, Zongshu Luo, Bruce M. McManus

**Affiliations:** UBC James Hogg Research Centre, Institute for Heart+Lung Health, Department of Pathology and Laboratory Medicine, University of British Columbia, Vancouver, British Columbia, Canada; Northwestern University Feinberg School of Medicine, United States of America

## Abstract

Growing evidence suggests the Wnt family of secreted glycoproteins and their associated signaling pathways, linked to development, are recapitulated during wound repair and regeneration events. However, the role of the Wnt pathway in such settings remains unclear. In the current study, we treated mouse fibroblasts with 250 ng/mL of recombinant Wnt3a for 72 hours and examined its affect on cell morphology and function. Wnt3a induced a spindle-like morphology in fibroblasts characterized by the increased formation of stress fibres. Wnt3a decreased the proliferation of fibroblasts, but significantly increased cell migration as well as fibroblast-mediated contraction of a collagen lattice. Wnt3a significantly increased the expression of TGF-β and its associated signaling through SMAD2. Consistent with this, we observed significantly increased smooth muscle α-actin expression and incorporation of this contractile protein into stress fibres following Wnt3a treatment. Knockdown of β-catenin using siRNA reversed the Wnt3a-induced smooth muscle α-actin expression, suggesting these changes were dependent on canonical Wnt signaling through β-catenin. Neutralization of TGF-β with a blocking antibody significantly inhibited the Wnt3a-induced smooth muscle α-actin expression, indicating these changes were dependent on the increased TGF-β signaling. Collectively, this data strongly suggests Wnt3a promotes the formation of a myofibroblast-like phenotype in cultured fibroblasts, in part, by upregulating TGF-β signaling through SMAD2 in a β-catenin-dependent mechanism. As myofibroblasts are critical regulators of wound healing responses, these findings may have important implications for our understanding of normal and aberrant injury and repair events.

## Introduction

Wound healing is a complex and dynamic process involving the interplay of many cellular and non-cellular components, typically culminating in the replacement of injured tissue with a fibrotic scar [1]. A number of soluble mediators released at the site of injury act as molecular cues that guide cellular responses during repair [2]. Evidence suggests the Wnt family of secreted glycoproteins and their associated signaling pathways, linked to development, are recapitulated during wound repair and regeneration events [3], [4]. However, the role of Wnt signaling in this setting remains unclear.

The Wnt signaling pathway is best recognized for its critical role in development of multi-cellular organisms [5], [6]. The Wnt family is comprised of 19 secreted glycoproteins that bind the Frizzled receptor and its co-receptor LRP5/6 (lipoprotein receptor-related proteins 5 or 6) to initiate an intracellular signaling cascade that controls the turnover of β-catenin (reviewed in [7]). In the absence of Wnt ligand, β-catenin is targeted for ubiquitin-mediated degradation by the 26S proteasome. Upon ligand stimulation, the canonical Wnt signaling pathway triggers a series of phosphorylation events that lead to the accumulation of cytosolic β-catenin, which then translocates to the nucleus where it binds the T-cell factor (TCF) or lymphoid enhancer binding factor (LEF) transcription factors to initiate transcription of target genes. As such, Wnt ligands can elicit a rapid and specific response in target cells.

In the current study, we examined the affect of Wnt3a, a canonical Wnt ligand, on fibroblast morphology and function. Our data suggests Wnt3a stimulates a spindle-like morphology in murine fibroblasts characterized by the increased expression of smooth muscle α-actin-positive stress fibres. These changes appear to be mediated, at least in part, by Wnt3a upregulating the expression of transforming growth factor (TGF)-β signaling through SMAD2 in a β-catenin-dependent manner. Collectively, this data suggests Wnt3a promotes the formation of a myofibroblast-like phenotype in cultured fibroblasts. As myofibroblasts are critical regulators of a wound healing response, these findings suggest a central role for Wnt signaling in regulating normal and aberrant injury and repair events.

## Materials and Methods

### Cell culture

Mouse embryonic fibroblasts (Clontech, product number 630914) were cultured in DMEM containing 10% FBS and 100 U/mL penicillin/streptomycin. Cells were maintained in a humidified incubator at 37°C with 5% CO_2_ and used for experiments between passages 6–12. Recombinant murine Wnt3a (Peprotech, product number 315–20) was added to cells at a concentration of 250 ng/mL for 72 hours prior to performing functional studies or collecting cells for analysis. Morphological changes were photographed with a Nikon 50i series upright microscope equipped with a digital camera. Confirmation of Wnt pathway activation was performed using a TOPFlash Reporter assay, as well as qPCR gene expression of axin2, as we have previously described [8], using predesigned primers to axin2 and β-actin (Applied Biosystems). In certain experiments, a TGF-β neutralizing antibody (Abcam, product number ab64715) was added during Wnt3a incubation at a concentration of 1 µg/mL. All experiments were performed in triplicate and repeated a minimum of 3 independent times.

### Cell proliferation and migration assays

Cell proliferation was measured by MTS assay (Promega) 72 hours after Wnt3a treatment. Migration was measured using the *in vitro* scratch wound assay, as previously described [9]. Briefly, cells were treated for 72 hours with Wnt3a and confluent monolayers were scratched using a dental device to create a cell-free area where migration could be measured.

### Collagen gel contraction assay

Twelve-well culture dishes were coated with 1% bovine serum albumin (BSA) and incubated for 1 hour at 37°C to create a non-stick surface that prevents gels from attaching to the dishes. Prior to performing contraction assays, fibroblasts were treated for 72 hours with Wnt3a. Cells were then trypsinized, counted and seeded into a 0.5 mg/mL Type I collagen solution (BD Biosciences, product number 354236) in growth media at a concentration of 1×10^5^ cells/mL. The collagen/cell suspension was vortexed, and 1 mL per well was added to the BSA-coated dishes and the solution was allowed to polymerize for 45 minutes at 37°C. Fresh growth media was added to the solidified collagen gels and plates were returned to the incubator. Collagen gel contraction was monitored over a period of 24 hours and the surface area of contracted gels was measured using Image-Pro Plus software (Media Cybernetics, Bethesda, USA).

### Western blotting

Cell lysates were collected in lysis buffer (10 mM HEPES (pH 7.4), 50 mM Na_4_P2O_7_, 50 mM NaF, 50 mM NaCl, 5 mM EDTA, 5 mM EGTA, 2 mM Na_3_VO_4_, and 1 mM phenylmethylsulfonyl fluoride, with 0.1% Triton X-100 and 10 µg/mL leupeptin) followed by centrifugation at high speed (14000 × g at 4°C for 10 minutes) to recover proteins. The protein concentration of samples was measured by a Bradford protein assay. Equal amounts of protein from each sample were separated with sodium dodecylsufate-polyacrylamide gel electrophoresis (SDS-PAGE; 10% polyacrylamide) and transferred to a nitrocellulose membrane. Membranes were blocked for 1 hour in 5% milk/TBS Tween 20 and incubated overnight at 4°C with primary antibody in 2.5% milk/TBST. Following 3 washes in TBST, secondary antibody (Santa Cruz biotechnology) at a concentration of 1∶2000 in 2.5% milk/TBST was added for 1 hour at room temperature. Antibody binding was visualized with the enhanced chemiluminescence detection system (Thermo Fischer Scientific). Images were captured with a Chemigenius2 system (Syngene, Frederick, USA) and band intensities were calculated with GeneTools software (Syngene).

Antibodies used were as follows: β-catenin (BD Biosciences, product number 610154), TGF-β (BD Biosciences, product number 555053), p-SMAD2 (Cell Signaling, product number 3108), SMAD2/3 (Cell Signaling, product number 3102), smooth muscle α-actin (Santa Cruz Biotechnology, product number sc-32251).

### Immunohistochemistry

Cells were fixed for 20 minutes in 3.7% formaldehyde, permeabilized with 0.1% triton X-100 for 20 minutes, blocked for 30 minutes with 1% BSA in PBS and incubated overnight at 4°C with the indicated primary antibody at a concentration of 1∶100 in 1% BSA. Following primary antibody, cells were washed with PBS and incubated with anti-mouse Alexa-fluor488 conjugated secondary antibody (Invitrogen) at a concentration of 1∶200 in 1% BSA for 1 hour at room temperature in the dark. To visualize f-actin, permeabilized cells were stained for 20 minutes with phalloidin conjugated to Alexa-fluor594 (Invitrogen). Cells were coverslipped with VectaShield mounting medium containing DAPI (Vector Laboratories) and images were captured using a Leica AOBS SP2 confocal microscope as we have previously described [10], [11].

### SiRNA transfections

Cells were seeded into 24-well culture dishes and siRNA transfection was performed using Oligofectamine (Invitrogen) as per the manufacturer's instructions. Control siRNA or mouse β-catenin siRNA (Santa Cruz Biotechnology, product numbers 37007 and 29210, respectively) were added to the cells at a concentration of 60 pmol/well for 24 hours prior to treating with Wnt3a. Cell lysates were harvested for Western blotting after 72 hours of Wnt3a treatment.

### Statistical analysis

Results are represented as the mean±standard deviation. Significant differences in treatment groups were determined using the unpaired Student's *t*-test. For all analyses, *P<0.05* was considered statistically significant.

## Results

### Wnt3a induces canonical Wnt signaling in mouse fibroblasts

**Figure 1:**
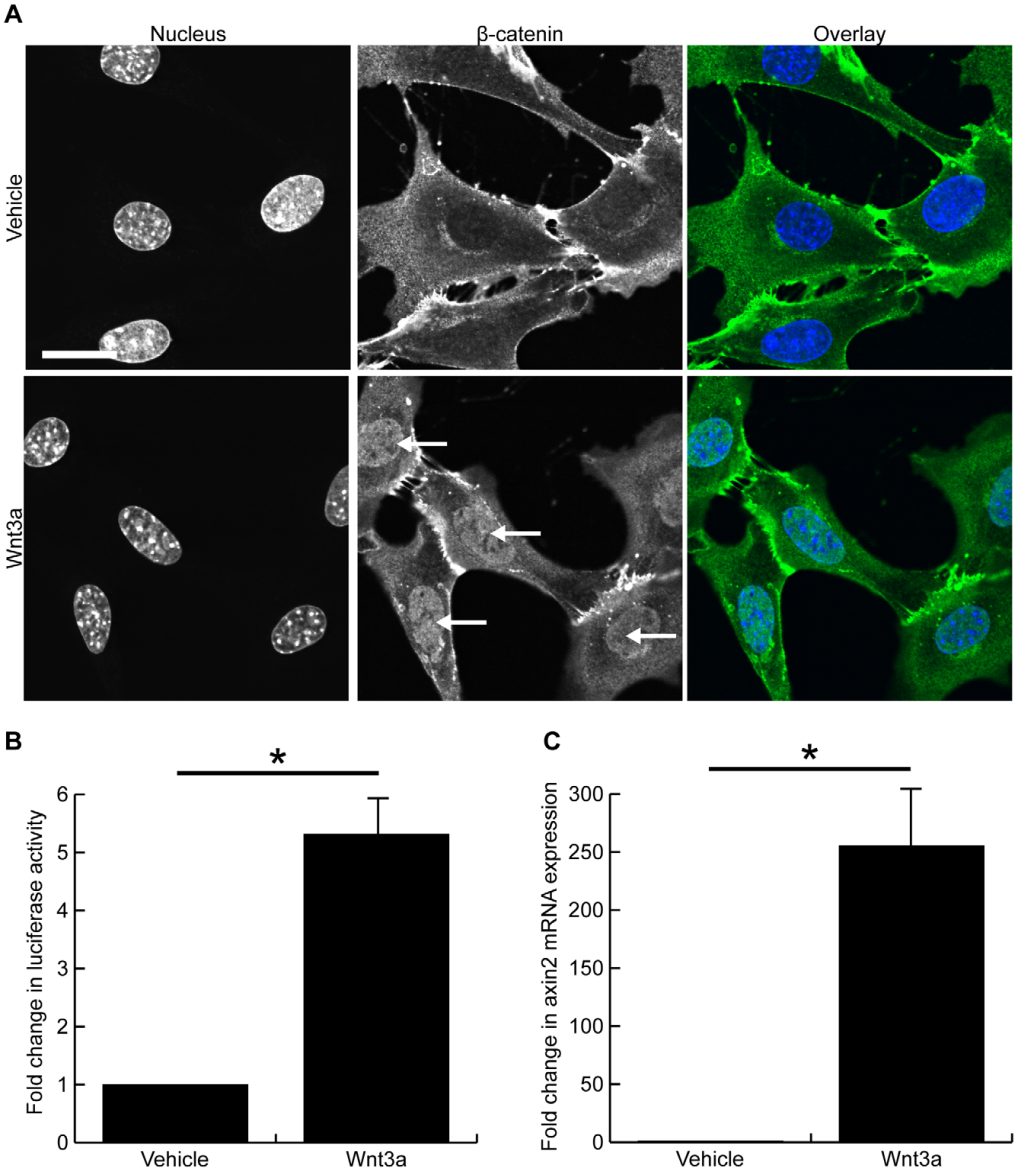
Wnt3a induces canonical Wnt signaling in mouse fibroblasts. Confocal images of fibroblasts treated for 24 hours with vehicle (top panels) or 250 ng/mL Wnt3a (bottom panels) and immunonstained for β-catenin (green) and nuclei (blue). Wnt3a treatment induced clear nuclear accumulation of β-catenin in murine fibroblasts (arrows). (B) TOPFlash reporter assay demonstrated Wnt3a significantly increased luciferase activity 5.3±1.6 fold after a 24 hour treatment (p<0.05). (C) Wnt3a treatment induced a 255±71 fold increase in the mRNA expression of axin2, a target of classical Wnt signaling (p<0.05). (Scale bar = 23.00 µm in A, * denotes p<0.05)

Mouse fibroblasts were treated for 24 hours with 250 ng/mL Wnt3a (or the vehicle control) to determine if recombinant Wnt3a induces nuclear accumulation of β-catenin. Immunohistochemistry demonstrated a strong nuclear signal for β-catenin in Wnt3a-treated fibroblasts while control cells remained negative for nuclear β-catenin (Figure 1A), suggesting Wnt3a activates canonical Wnt signaling. To confirm activation of canonical Wnt signaling by Wnt3a treatment, cells were transfected with a TOPFlash reporter construct prior to Wnt3a treatment, and a luciferase assay demonstrated that Wnt3a activated the TOPFlash reporter 5.3±1.6 fold after a 24 hour treatment (Figure 1B, p<0.05). Further, mRNA expression of axin2, an early immediate target of canonical Wnt signaling, was measured after 24 hours of Wnt3a treatment. Wnt3a induced a 255±71 fold increase in axin2 mRNA expression compared with vehicle treated cells (Figure 1C, p<0.05).

### Wnt3a alters the morphology of mouse fibroblasts

**Figure 2:**
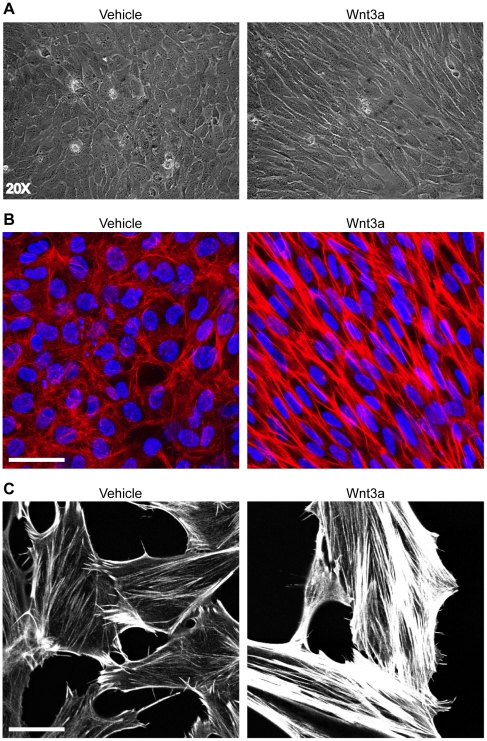
Wnt3a induces a spindle-like morphology with increased stress fibre formation after 72 hours of treatment. (A) Light microscope images of mouse fibroblasts that had been treated for 72 hours with vehicle (left panel) or 250 ng/mL Wnt3a (right panel). Wnt3a treatment induced a spindle-like morphology in fibroblasts. (B) Confocal images of vehicle-treated (left panel) or Wnt3a-treated (right panel) fibroblasts immunostained for f-actin (red) and nuclei (blue) showing the increased formation and parallel organization of stress fibres following 72 hours Wnt3a treatment. (C) Low density culture of vehicle-treated (left panel) or Wnt3a-treated (right panel) fibroblasts highlights the increased formation of stress fibres seen after Wnt3a treatment. (Scale bars = 47.00 µm in B, 23.00 µm in C)

Wnt3a induced a marked change in fibroblast morphology after 72 hours of treatment. The Wnt-treated cells appeared spindle-shaped and organized into parallel sheets as visualized by light microscopy (Figure 2A). Consistent with this, confocal microscopy showed Wnt3a-treated cells appeared larger and had altered cytoskeletons characterized by dramatically increased stress fibre formation (Figure 2B). The increased formation of stress fibres in Wnt3a-treated fibroblasts is best visualized in low density cultures of the cells (Figure 2C).

### Wnt3a inhibited fibroblast proliferation, but increased cell migration and contraction of collagen gels

**Figure 3:**
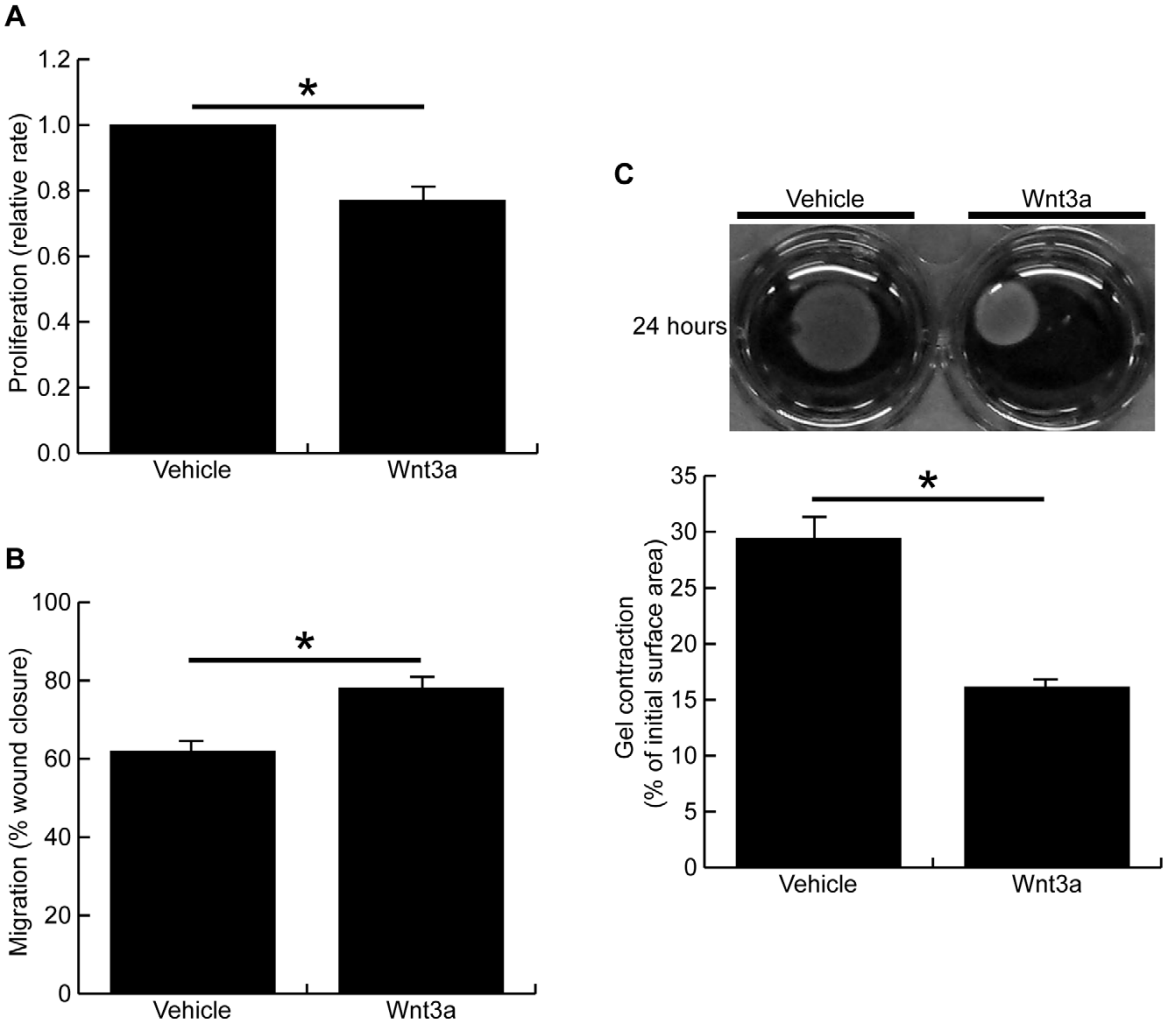
Wnt3a inhibits cell proliferation, but increases cell migration and contraction after 72 hour treatment. (A) Cell proliferation was measured after 72 hours of treatment with Wnt3a or vehicle. Wnt-treated cells grew at 77.4±4.5% the rate of vehicle treated cells (p<0.05). (B) Cells were treated for 72 hours with Wnt3a or vehicle, and then a scratch wound assay was performed to measure cell migration. Wnt-treated cells closed the scratch wound at a significantly faster rate than vehicle-treated cells, as measured 48 hours after injury (78.1±2.1% vs 61.9±3.8%, p<0.05). (C) Cells were treated for 72 hours with Wnt3a or vehicle and then a fibroblast-populated collagen lattice contraction assay was performed. Images of contracted gels taken at 24 hours are shown along with the quantified surface areas of contracted gels. Wnt3a treatment significantly increased the fibroblast-mediated contraction of collagen gels (16.1±0.6% vs 29.4±1.3% of initial surface area, p<0.05). (* denotes p<0.05)

Cell proliferation was observed to be significantly decreased after 72 hours Wnt3a treatment (Figure 3A, proliferation rate: 77.4±4.5% of vehicle-treated cells, p<0.05). In contrast, Wnt3a significantly increased cell migration as measured by *in vitro* scratch wound assay (Figure 3B, 78.1±2.1% vs 61.9±3.8%, p<0.05). Fibroblast contraction, as measured by a fibroblast-populated collagen lattice contraction model, was also found to be significantly increased following the 72 hour Wnt3a treatment (Figure 3C, 16.1±0.6% vs 29.4±1.3% of initial gel area, p<0.05).

### Wnt3a increases TGF-β expression, SMAD2 phosphorylation and smooth muscle α-actin expression

**Figure 4:**
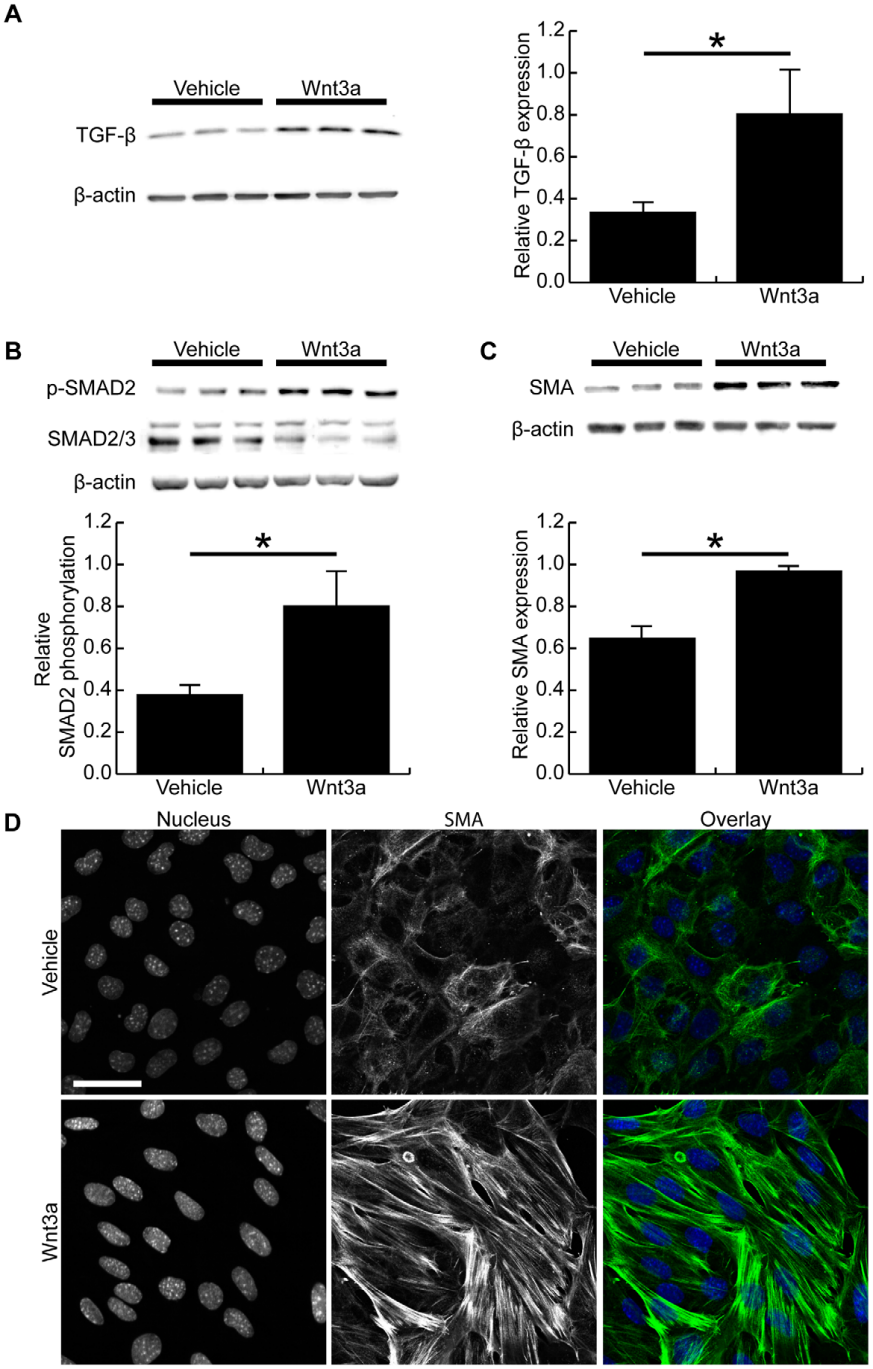
Wnt3a increases TGF-β expression, SMAD2 phosphorylation and smooth muscle α-actin expression. (A) Representative Western blot of TGF-β expression in vehicle-treated and Wnt3a-treated fibroblasts after 72 hours. Densitometry showed TGF-β expression to be significantly increased after Wnt3a treatment (p<0.05). (B) Western blot of SMAD2 phosphorylation after 72 hours of vehicle or Wnt3a treatment. Densitometry showed Wnt3a significantly increased SMAD2 phosphorylation at 72 hours. (C) Western blot of smooth muscle α-actin expression in vehicle-treated or Wnt3a-treated cells. Wnt3a-treatment significantly increased the expression of smooth muscle α-actin expression in mouse fibroblasts, as measured by densitometry (p<0.05). (D) Confocal images of fibroblasts immunostained for smooth muscle α-actin (green) and nuclei (blue). Wnt3a-treated fibroblasts had clearly visible smooth muscle α-actin positive stress fibres while the vehicle-treated cells did not display expression of smooth muscle α-actin in their stress fibres. (Scale bar = 47.00 µm in D, * denotes p<0.05)

Morphologically and functionally, our data suggests Wnt3a stimulates a myofibroblast-like phenotype in cultured fibroblasts. We next examined whether Wnt3a alters the TGF-β signaling axis in these cells. Western blot demonstrated expression of TGF-β to be upregulated after 72 hours Wnt3a treatment, and densitometry showed this change to be significant (Figure 4A). Consistent with this, SMAD2 phosphorylation, a downstream signaling target of TGF-β, was shown to be significantly increased following Wnt3a treatment (Figure 4B). Smooth muscle α-actin, the most commonly used marker of myofibroblast differentiation, was also found to be significantly upregulated by Wnt3a (Figure 4C). Immunohistochemistry and confocal microscopy confirmed this, and showed the spindle-shaped fibroblasts displayed clearly visible smooth muscle α-actin-positive stress fibres following Wnt3a treatment (Figure 4D).

### Wnt3a-induced smooth muscle α-actin expression is dependent on β-catenin

**Figure 5:**
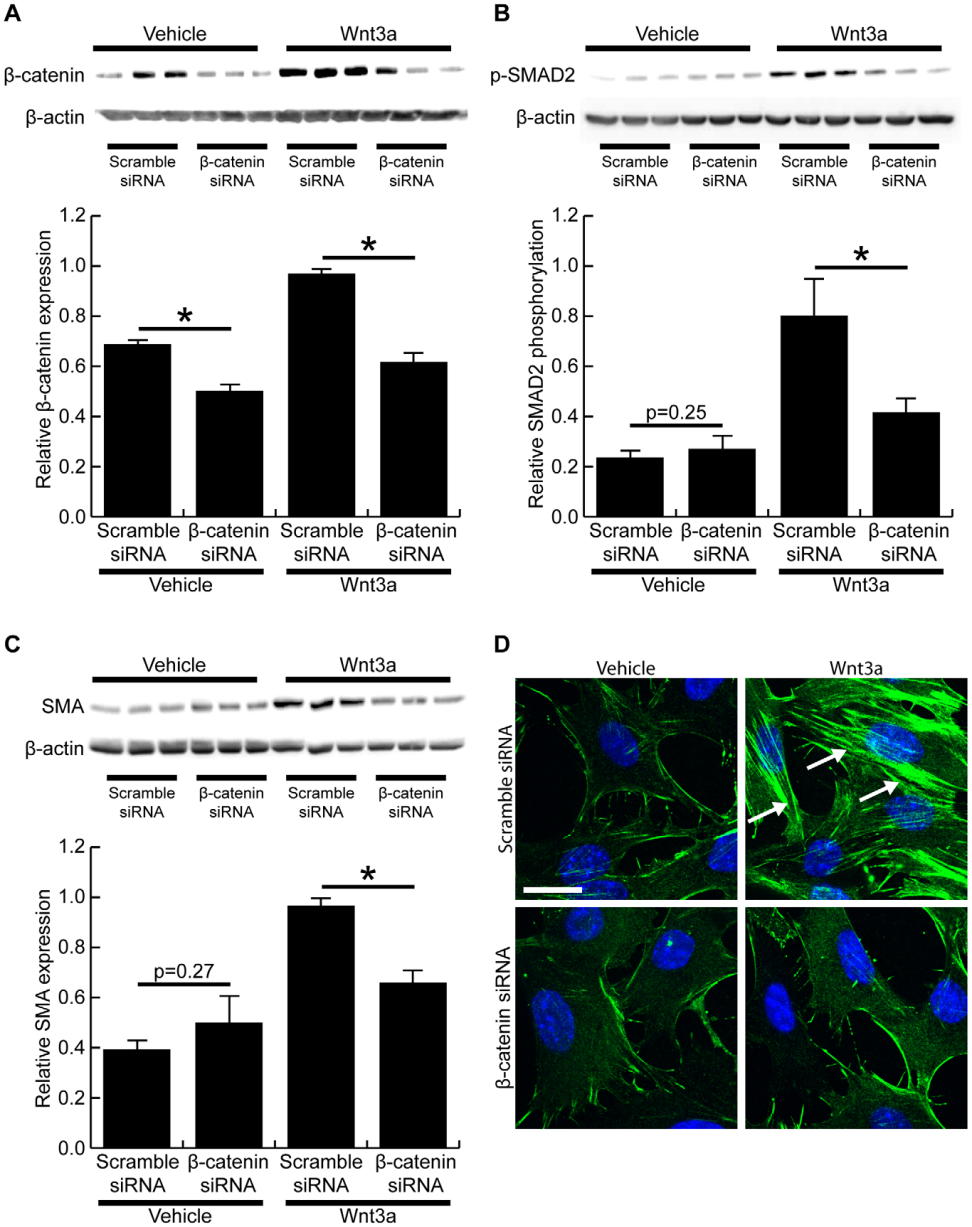
Wnt3a-induced change in cell phenotype is dependent on β-catenin. (A) Western blot demonstrated β-catenin siRNA significantly decreased β-catenin expression in vehicle- and Wnt-treated fibroblasts when compared to a scrambled siRNA. (B) Western blot showed knock down of β-catenin expression significantly inhibited the Wnt3a-induced SMAD2 phosporylation (p<0.05). No difference in SMAD2 phosporylation was detected in vehicle treated cells (p = 0.25). (C) Western blot of smooth muscle α-actin expression demonstrated that β-catenin siRNA significantly decreased smooth muscle α-actin expression in Wnt3a-treated fibroblasts (p<0.05). No significant difference was seen in the vehicle-treated cells (p = 0.27). (D) Immunohistochemistry showed Wnt3a promoted smooth muscle α-actin stress fibre formation in control siRNA transfected cells (green, arrows), but β-catenin siRNA completely inhibited the Wnt3a-induced smooth muscle α-actin expression. Cell nuclei are stained blue with DAPI. (Scale bar = 23.00 µm in D, * denotes p<0.05)

To determine if the altered cell phenotype induced by Wnt3a was dependent on β-catenin, we used siRNA to knock down β-catenin expression prior to treating cells with Wnt3a. Western blot showed siRNA significantly decreased β-catenin expression in both vehicle- and Wnt3a-treated cells (Figure 5A). Knock down of β-catenin resulted in a 48.5±8.4% decrease in Wnt3a-induced axin2 mRNA expression (p<0.05, data not shown), suggesting β-catenin siRNA significantly inhibited signaling through the canonical Wnt pathway. In the absence of Wnt3a, no change in SMAD2 phosphorylation was observed in cells transfected with scrambled or β-catenin siRNA. Upon stimulation with Wnt3a, however, β-catenin siRNA significantly inhibited the Wnt3a-induced SMAD2 phosphorylation (Figure 5B). The decreased SMAD2 phosphorylation was associated with significantly decreased smooth muscle α-actin expression in Wnt3a-treated cells that had been transfected with β-catenin siRNA (Figure 5C). No change in smooth muscle α-actin expression was observed in vehicle-treated cells. Immunohistochemistry and confocal microscopy confirmed that β-catenin knockdown inhibited the Wnt3a-induced smooth muscle α-actin expression in mouse fibroblasts (Figure 5D).

### Wnt3a-induced change in cell phenotype is dependent on TGF-β expression

**Figure 6:**
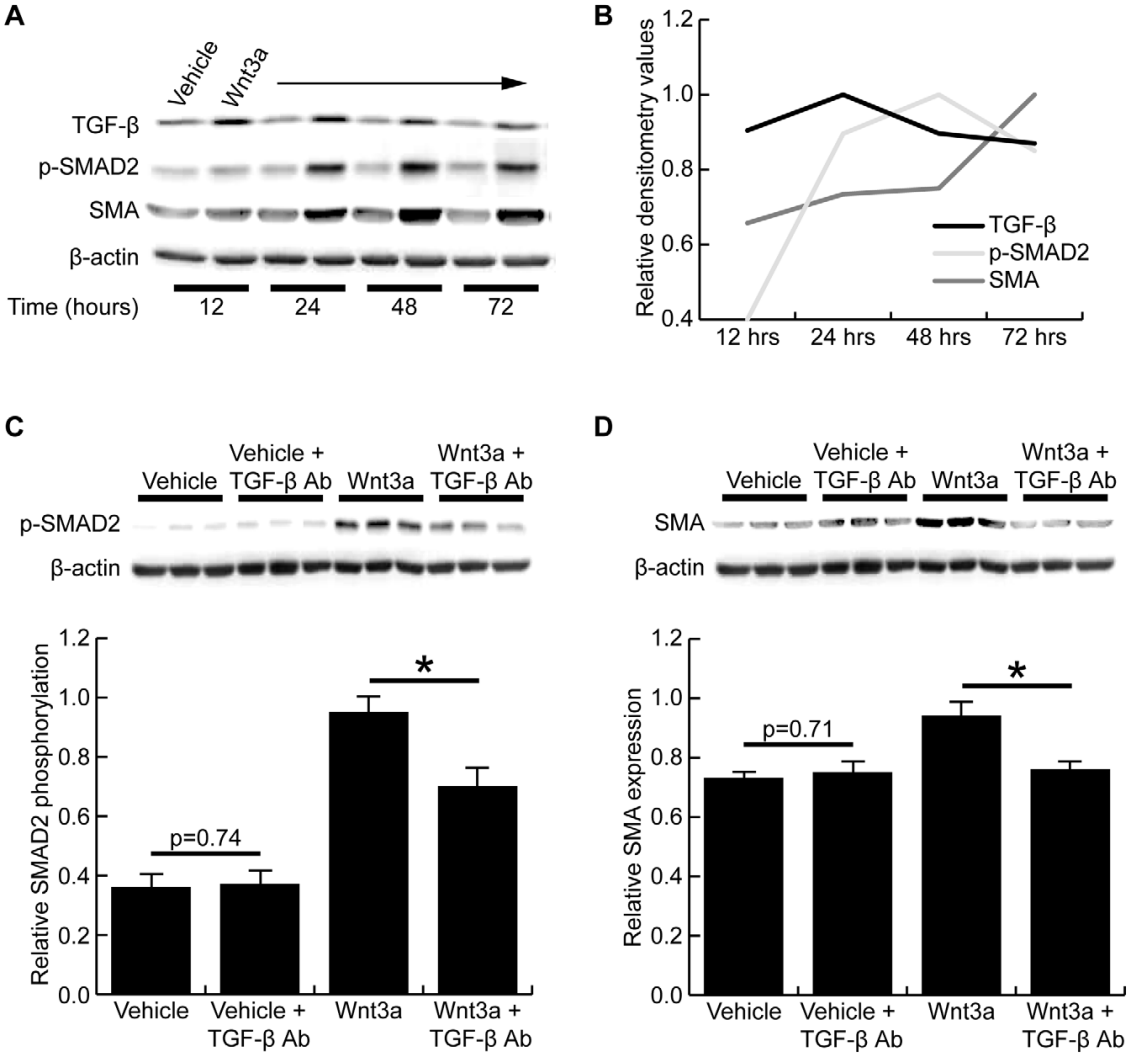
Wnt3a-induced change in cell phenotype is dependent on TGF-β expression. (A) Representative Western blots of vehicle- and Wnt3a-treated fibroblasts showing TGF-β expression, SMAD2 phosphorylation, and smooth muscle α-actin expression at 12, 24, 48, and 72 hours of treatment. (B) Graphical representation of the densitometry results for the blots in A shows, in a sequential manner, that TGF-β expression peaks between 12 and 24 hours, followed by SMAD2 phosphorylation peaking between 24 and 48 hours, which is then followed by smooth muscle α-actin expression peaking after 72 hours of treatment. (C) Western blot of SMAD2 phosphorylation in fibroblasts treated with or without Wnt3a and a TGF-β neutralizing antibody. Densitometry demonstrated the TGF-β neutralizing antibody significantly inhibited Wnt3a-induced SMAD2 phosphorylation (p<0.05). No change was seen in the vehicle-treated cells (p = 0.74). (D) Western blot of smooth muscle α-actin expression in fibroblasts treated with or without Wnt3a and the TGF-β neutralizing antibody. Densitometry confirmed TGF-β neutralization significantly inhibited the Wnt3a-induced smooth muscle α-actin expression (p<0.05). No change was seen in vehicle-treated cells (p = 0.71). (* denotes p<0.05)

To better characterize the role of TGF-β signaling in Wnt3a-treated fibroblasts, a time course experiment was performed over 72 hours. Representative Western blots are shown in Figure 6A and the relative densitometry values are plotted over time in Figure 6B. Wnt3a treatment led to a rapid induction of TGF-β expression, which was highest between 12 and 24 hours after treatment. Phosphorylation of SMAD2 appeared highest between 24 and 48 hours, which was followed by the strongest expression of smooth muscle α-actin after 72 hours of treatment, indicating a sequential activation of this pathway following Wnt3a treatment. To determine if the Wnt3a-induced SMAD2 phosphorylation and smooth muscle α-actin expression were dependent on TGF-β expression, a neutralizing antibody to TGF-β was added during Wnt3a treatment. TGF-β neutralization significantly inhibited both the Wnt3a-induced phosphorylation of SMAD2 (Figure 6C) and smooth muscle α-actin expression (Figure 6D). No change in SMAD2 phosphorylation or smooth muscle α-actin expression was seen in the vehicle-treated cells.

## Discussion

In this study, we have shown that Wnt3a alters the phenotype of mouse fibroblasts. Structurally, Wnt3a induced a spindle-shaped morphology characterized by increased expression and incorporation of smooth muscle α-actin into stress fibres. Functionally, Wnt3a inhibited fibroblast proliferation, but increased cell migration and contraction. These changes were mediated, at least in part, by Wnt3a-induced TGF-β expression and signaling through SMAD2 in a β-catenin-dependent mechanism. Collectively, this data suggests Wnt3a stimulates the formation of a myofibroblast-like phenotype in cultured fibroblasts.

Our data are consistent with recent studies suggestive of an interaction between Wnt/β-catenin and TGF-β/SMAD signaling in controlling gene transcription and cell phenotype [12], [13], [14]. A recent report has demonstrated that Wnt3a controls transcriptional regulation of SM22α in mesenchymal cells via convergence with TGF-β/SMAD signaling at a novel regulatory element in the SM22α promoter [13]. SM22α is a calponin-like protein that exhibits a similar expression pattern as smooth muscle α-actin [15], both of which are smooth muscle cell contractile proteins commonly viewed as markers of an activated myofibroblast phenotype [16]. In a separate study that documented the gene expression profile induced by Wnt3a in fibroblasts, TGF-β was identified as one of the genes upregulated more than two fold after a 6 hour treatment [17]. Consistent with this, Wnt3a was found to stimulate TGF-β and collagen I mRNA expression in cultures of fetal and post-natal fibroblasts [14]. Our data add to this story by showing Wnt3a stimulates TGF-β protein expression and activation of its downstream signaling, culminating in increased smooth muscle α-actin expression. In another study, Laeremans *et al* showed overexpression of the Frizzled 1 receptor in combination with Wnt3a treatment stimulated the expression of myofibroblast markers in cardiac fibroblasts, changes that occurred in a β-catenin-independent pathway [18]. Interestingly, Wnt3a treatment alone was actually found to decrease the expression of smooth muscle α-actin in their study. The cause of such discrepancy is not clear at present. However, taken together, these studies support a role for Wnt3a in modifying cell phenotype, with our data strongly suggesting Wnt3a promotes a myofibroblast-like phenotype in cultured fibroblasts.

Growing evidence points to an active role for Wnt signaling in normal wound repair and in a number of human diseases. Increased canonical Wnt signaling has been observed during cutaneous wound repair [14], [19], [20], but is also well recognized as a contributor to a multitude of malignant disorders [21], [22], [23], as well as hypertrophic scarring [24], aberrant blood vessel remodeling [25], pulmonary fibrosis [26] and aging [27], among others. After tissue injury, fibroblasts differentiate into contractile and secretory myofibroblasts that participate in the synthesis and remodeling of granulation tissue during repair [28]. However, these myofibroblasts can severely impair organ function when contraction and extracellular matrix secretion become excessive [29]. Moreover, myofibroblasts present in the stroma reaction of epithelial tumors may promote the progression of cancer invasion [29], [30]. TGF-β is a known and potent inducer of myofibroblast differentiation [31], [32], however the regulation of TGF-β expression remains relatively understudied. The finding that Wnt3a upregulates TGF-β expression and stimulates smooth muscle α-actin expression provides a link between Wnt signaling and myofibroblasts in wound repair and disease. If Wnt3a also upregulates TGF-β expression and myofibroblast differentiation *in vivo*, the Wnt signaling pathway may be shown as a critical regulator of the wound healing response. More work will be needed to determine how manipulating the Wnt pathway alters injury and repair events *in vivo*.

Wnt proteins are believed to signal through three distinct pathways, of which the canonical Wnt/β-catenin cascade is the best understood. The other pathways include the noncanonical planar cell polarity pathway and the Wnt/Ca^2+^ pathway [6]. By using siRNA, we were able to demonstrate that knocking down β-catenin expression reversed the Wnt3a-induced smooth muscle α-actin expression, suggesting these changes were mediated by canonical Wnt signaling through β-catenin. In addition, we demonstrated that Wnt3a also upregulated TGF-β expression. Previous reports have identified TGF-β as one of the genes whose mRNA expression is rapidly induced by Wnt3a treatment [14], [17]. Thus, there appears to be sufficient evidence to suggest TGF-β is a target of canonical Wnt signaling. To our knowledge, however, there are no published reports that have examined whether TGF-β gene transcription is regulated by Wnt signaling or whether its promoter contains functionally important TCF/LEF binding sites. It will be important to determine whether TGF-β is one of a growing list of direct targets genes for Wnt signaling, as this information might provide new therapeutic targets for controlling TGF-β levels in disease settings.

In summary, we provide data on a novel role for Wnt3a in stimulating myofibroblast differentiation in cultured fibroblasts. Our data suggest Wnt3a treatment promotes a contractile and migratory fibroblast phenotype that is characterized by increased expression of smooth muscle α-actin. These changes appear to be mediated by increased expression of TGF-β and signaling through SMAD2 in a β-catenin-dependent manner. As myofibroblasts play a central role in normal and aberrant injury and repair events, this data suggests Wnt3a may be critically involved in the wound healing response.
